# Thermodilution vs estimated Fick cardiac output measurement in an elderly cohort of patients: A single-centre experience

**DOI:** 10.1371/journal.pone.0226561

**Published:** 2019-12-20

**Authors:** Karl-Patrik Kresoja, Alessandro Faragli, Dawud Abawi, Oliver Paul, Burkert Pieske, Heiner Post, Alessio Alogna

**Affiliations:** 1 Department of Internal Medicine and Cardiology, Campus Virchow Klinikum (CVK), Charité–University Medicine, Berlin, Germany; 2 Berlin Institute of Health, Berlin, Germany; 3 German Cardiovascular Research Centre (DZHK), partner site Berlin, Germany; 4 German Heart Center Berlin, Berlin, Germany; 5 Department of cardiology and angiology, St. Marien-Hospital Mülheim, Mülheim, Germany; Scuola Superiore Sant'Anna, ITALY

## Abstract

**Aims:**

Patients referred to the cath-lab are an increasingly elderly population. Thermodilution (TD, gold standard) and the estimated Fick method (eFM) are interchangeably used in the clinical routine to measure cardiac output (CO). However, their correlation in an elderly cohort of cardiac patients has not been tested so far.

**Methods:**

A single, clinically-indicated right heart catheterization was performed on each patient with CO estimated by eFM and TD in 155 consecutive patients (75.1±6.8 years, 57.7% male) between April 2015 and August 2017. Whole Body Oxygen Consumption (VO2) was assumed by applying the formulas of LaFarge (LaF), Dehmer (De) and Bergstra (Be). CO was indexed to body surface area (Cardiac Index, CI).

**Results:**

CI-TD showed an overall moderate correlation to CI-eFM as assessed by LaF, De or Be (r^2^ = 0.53, r^2^ = 0.54, r^2^ = 0.57, all p < .001, respectively) with large limits of agreement (-0.64 to 1.09, -1.07 to 0.77, -1.38 to 0.53 l/m^2^/min, respectively). The mean difference of CI between methods was 0.22, -0.15 and -0.42 (all p<0.001 for difference to TD), respectively. A rate of error ≥20% occurred with the equations by LaF, De or Be in 40.6%, 26.5% and 36.1% of patients, respectively. A CI <2.2 l/m^2^min was present in 42.6% of patients according to TD and in 60.0%, 31.0% and in 16.1% of patients according to eFM by the formulas of LaF, De or Be.

**Conclusion:**

Although CI-eFM shows an overall reasonable correlation with CI-TD, the predictive value in a single patient is low. CI-eFM cannot replace CI-TD in elderly patients.

## Introduction

Cardiac output (CO) is a key haemodynamic parameter for guiding therapy in patients with heart failure, pulmonary hypertension (PH) and valvular heart disease. CO is usually measured by the thermodilution- (TD) or the Fick-method (FM). The FM relies on the direct measurement of the whole-body oxygen consumption (VO_2_). In clinical practice, VO_2_ is often estimated rather than actually measured (estimated Fick method, eFM), which results in a certain margin of error. One of the following three published empirical formulas by LaFarge (LaF)[1], Dehmer (De)[2] and Bergstra (Be)[3] is commonly used to estimate VO_2._ The formulas predict VO_2_ at rest, based on a certain combination of the following variables: body surface area (BSA),[1–3] age,[1,3] gender[1,3] and heart rate[1].

As a major drawback, these formulas were derived and validated in paediatric cohorts of patients [1,4,5], since invasive CO measurements were usually performed in conditions of PH or congenital heart failure. However, as the number of elderly patients affected by cardiovascular disease keeps rising steadily in industrialised countries, the need for tailored medical approaches for this growing, yet under-investigated subset of patients is needed.[6,7] With the increasing number both of patients undergoing transcatheter aortic valve implantations (TAVI) and patients diagnosed with atypical pulmonary hypertension (a disease typically appearing in the aged population), more elderly patients need an invasive haemodynamic assessment. [8–10]

This study aimed to investigate the correlation of the eFM-CO and TD-CO in an elderly real-world cohort of all-comers patients. Also, we investigated variables leading to a mismatch in the estimated and the measured CO, like severe valve regurgitation, low cardiac output and estimated body fat.

## Materials and methods

The study protocol was conducted in accordance with the amended Declaration of Helsinki and was approved by the local independent Ethic Committee of the Charité University Clinic, Germany (EA2/242/18). Data were analyzed retrospectively and anonymised.

### Study design and patient cohort

Consecutive patients ≥60 years undergoing elective clinically indicated right-heart catheterization were included in this retrospective single-centre study at the Charité–Campus Virchow Klinikum, Department of Internal Medicine and Cardiology, Germany. The complete baseline data on clinical, electrocardiographic, echocardiographic, haemodynamic and laboratory parameters were obtained using a standardised and anonymised questionnaire case report form.

Patients were excluded for the following reasons: a) missing baseline data, b) missing variables to determine CO c) presence of a relevant shunt Qp/Qs >1.5 and d) fever or other systemic illnesses increasing VO_2_ e) sedation with >2 mg Lorazepam, the use of a comparable tranquilizer or any other kind of sedatives.

The primary objective of this study was to determine cardiac index (CI) by the two methods of TD and eFM. Since there was no difference in the results in either using CI or CO, only the CI data is presented. VO_2_ was calculated based on the formulas of LaFarge[1], Dehmer[2] and Bergstra[3]. The Fick formula was used to calculate VO_2_ based on CO as measured by TD. BSA was calculated using the formulas by Du Bois and Du Bois[11]. Cardiac catheterization and haemodynamic assessment were performed accordingly to current recommendations in a supine position.[12] Haemodynamics were assessed by original pressure tracings by two independent authors (K-P. K., A.A.). TD was performed with an *Edwards Lifescience Vigilance* II^™^ monitor. In patients with sinus rhythm at least three, whereas in patients with atrial fibrillation or other cardiac arrhythmias at least five repetitive measurements were performed, and the results presented as mean values.

Blood-gas analysis was performed using an ABL800 FLEX blood-gas Analyser^™^ (Radiometer, Denmark). If concomitant left heart catheterization was performed, arterial oxygen saturation (SaO_2_) was measured by blood-gas analysis. Otherwise, SaO_2_ was obtained through pulse oximetry. In the case of two blood-gas analysis samples, the mean haemoglobin of venous and arterial samples was used for the calculation of eFM. Body fat was calculated using a calliper, as proposed by Jackson and Pollock’s method. [13,14]

### Statistical analysis

Categorical variables are expressed as absolute numbers or in percentage and were compared, as appropriate, using Fisher's exact test or a Chi-squared test. Continuous variables did not follow a normal distribution when tested with the modified Kolmogorov-Smirnov test (Lilliefors test); these variables are expressed as mean with the corresponding standard deviation (± SD) and were compared using the unpaired Mann-Whitney U-test.

Pearson’s and Spearmen’s correlation coefficients (r) and coefficients of determination (r^2^), as appropriate, were calculated for comparative purposes while confidence intervals of the correlation coefficient were calculated using Fisher’s Z-transformation. The agreement between methods was analysed as described by Bland and Altman.[15]

A two-sided significance level of α 0.05 was defined as appropriate to indicate statistical significance. Statistical analyses were performed using the SPSS software (IBM Corp. Released 2016. IBM SPSS Statistics for Windows, Version 24.0. Armonk, NY: IBM Corp.) and GraphPad Prism (version 7.00 for Windows, GraphPad Software, La Jolla California USA).

## Results and discussion

### Study sample characteristics

Data on 155 patients (mean age 75.1 ± 6.8, 57.7% male) undergoing the investigation between April 2015 and August 2017 were analysed. The baseline characteristics and comorbidities are presented in Table 1, while baseline haemodynamic- and blood-gas-results are presented in S2 Table. Valvular heart disease (76.8%) and evaluation for PH (21.2%) were the leading indications for right-heart catheterization. Two patients (1.2%) underwent cardiac catheterization for suspected constrictive pericarditis, which was excluded in both cases.

**Table 1:** Baseline characteristics.

Baseline Data	n = 155
Age, years	75.1 ± 6.8
Male	90 (57.7)
Height (cm)	169.8 ± 10.5
Weight (kg)	79.0 ± 17.1
BSA DuBois (m^2^)	1.90 ±0.23
**BMI (kg/m^2^)**	27.3 ± 5.2
BMI <18	3 (1.9)
BMI 18–25	52 (33.5)
BMI 25–30	52 (33.5)
BMI >30	48 (31.0)
Estimated Body fat (%)	28.7 ± 12.0/126
**Lab**	
Creatinine (mg/dL)	1.50 ± 1.37
**GFR (ml/min)**	
GFR <30 ml/min/1.73m^2^	23 (14.9)
GFR <60 ml/min/1.73m^2^	79 (51)
Haemodialysis	9 (5.8)
Diabetes mellitus	64 (41.2)
**Echocardiography**	
**LVEF (%)**	48.6 ± 11.6
LVEF <40%	31 (20.0)
LVEF 40–50%	21 (13.5)
LVEF ≥50%	103 (66.5)
Severe aortic stenosis	52 (33.5)
Severe aortic regurgitation	1 (0.6)
Severe mitral stenosis	4 (2.6)
Severe mitral regurgitation	39 (25.1)
Severe pulmonary stenosis	1 (0.7)
Severe pulmonary regurgitation	0
Severe tricuspid stenosis	0
Severe tricuspid regurgitation	23 (14.8)

Abbreviations: BSA denominates body surface area; BMI, body mass index; GFR, glomerular filtration rate and LVEF left ventricular ejection fraction.

Overall 20.0% of the study population suffered from heart failure with reduced ejection fraction (HFrEF), 13.5% had heart failure with mid-range reduced ejection fraction (HFmrEF), and 66.5% had a preserved left ventricular ejection fraction (HFpEF). With regard to the HFpEF population, all but two patients had symptoms or signs of heart failure and signs of either structural or valvular heart disease. Severe aortic stenosis and mitral regurgitation were the most frequent valvular diseases (33.5 and 25.1%, respectively) followed by severe tricuspid regurgitation (14.8%). Body-mass-index (BMI) was almost equally distributed between a range of 18–25, 25–30 and > 30 kg/m^2^, while a BMI of <18 kg/m^2^ was only observed in 1.9% of the population. Estimated mean body fat of the overall population was 28.7 (± 12.0) %.

### Correlation between thermodilution- and estimated Fick-method

CI assessed by the eFM differed significantly from CI-TD for all three empiric formulas as displayed in Table 2, being lower for LaF und higher for De and Be. The coefficient of variation for repeated measurements of TD-CO was 7.8%. The correlation between CI-TD and CI-eFM was moderate for all three formulas (r^2^ 0.53, 95% CI 0.42–0.63; r^2^ 0.54, 95% CI 0.42–0.64, r^2^ 0.57, 95% CI 0.45–0.66, for LaF, De and Be respectively, all p < .001). Interestingly the correlation between the VO_2_-TD and the estimated VO_2_ was weaker than the one between CI-TD and CI-eFM (S3 Table).

**Table 2:** 

Method	Cardiac Output (l/min)	p*	Cardiac Index	p*	VO_2_	p*
Thermodilution	4.49 ± 1.33	NA	2.36 ± 0.56	NA	226 ± 52	NA
Lafarge	4.08 ± 1.27	<0.001	2.14 ± 0.56	<0.001	204 ± 42	<0.001
Dehmer	4.76 ± 1.32	<0.001	2.51 ± 0.63	<0.001	237 ± 29	<0.001
Bergstra	5.30 ± 1.54	<0.001	2.78 ± 0.70	<0.001	263 ± 39	<0.001

Abbreviations: VO_2_ denominates oxygen consumption.

*p for estimated cardiac output (LaFarge, Dehmer, Bergstra, respectively) vs measured cardiac output (thermodilution).

### Agreement between TD-method and eFM

**Fig 1:**
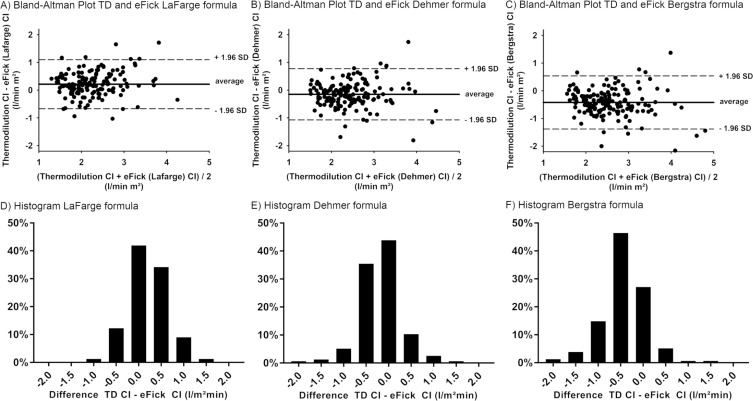
**a-f. Bland-Altman Plots and histograms comparing measured and estimated CI by the formulas of LaFarge, Dehmer and Bergstra.** a-c) Bland Altman Plots comparing measured and estimated CI by the formulas of LaFarge, Dehmer and Bergstra, respectively; d-e) Histograms comparing the absolute differences between measured and estimated CI by the formulas of LaFarge, Dehmer and Bergstra, respectively. Abbreviations: CI denominates cardiac index; eFick, estimated Fick method and SD, standard deviation.

**Fig 2:**
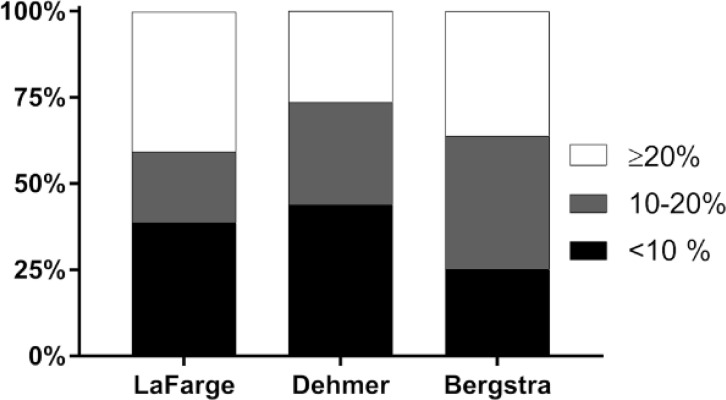
Relative difference between indirect Fick method and thermodilution assessed cardiac index. Black bars indicate the percentage of patients with an error between cardiac index measurements <10%, grey bars indicate the percentage of patients with an error between cardiac index measurements 10–20%, the white bar indicates the percentage of patients with an error between cardiac index measurements >20%.

Fig 1 displays the Bland-Altman plots on the agreement between CI-TD and CI-eFM. CI-eFM shows large limits of agreement when compared to the measured CI by the TD-method. The broadest limits of agreement were seen with the Be formula (-1.38 to 0.53), followed by the formula proposed by De (-1.07 to 0.77), while the narrowest limits of agreement were achieved using the LaF formula (-0.64 to 1.09). In order to further characterize the agreement of CI-TD and CI-eFM, we divided patients into 3 groups with a relative difference of ≤ 10%, 10–20% or ≥ 20%. From the three established formulas, the De formula (43.9%) had the highest number of patients with an error rate ≤10% and the lowest number with an error rate ≥ 20% (26.5%), as shown in Fig 2.

### Potential confounders of CI measurement and estimation in the study sample

The correlation between CI-TD and CI-eFM in patients with none- or mild and moderate valve regurgitation did not differ relevantly. The mean difference between CI-TD and the CI-eFM as assessed by La was lower in patients with severe tricuspid regurgitation (0.048 ± 0.447 vs 0.256 ± 0.440, p = 0.028), while it did not differ when CI-eFM was assessed by De or Be. Excluding patients with severe tricuspid regurgitation improved the coefficient of determination of the correlation between CI-TD and CI-eFM (S4 Table). No tests were performed for patients affected by severe aortic- or pulmonary regurgitation due to the limited number of patients.

States of low cardiac output (Cardiac index <2.2 l/min/m^2^) were associated with lower CI values when comparing the TD-method to the eFM with the formulas of LaF and De (0.061 ± 0.376 vs. 0.345 ± 0.455, p < .001 and -0.262 ± 0.388 vs. -0.068 ± 0.512, p = 0.008, respectively).

Obesity (BMI >35 kg/m^2^) and a body fat percentage of more than 30% did not affect the correlation between CI-TD and CI-eFM.

Predictors of a difference larger than 20% between estimated and measured CI are presented in S5 Table. Briefly, predictors for a difference equal or greater to 20% were female sex and age ≥78 years (optimised cut-derived from ROC analysis) with LaF formula, as well as a cardiac Index <2.2 l/min/m^2^ with the Be formula.

Primarily because of the increased life expectancy, patients referred to the cath-lab are an increasingly aging population. TD, as the gold standard, and the eFM are interchangeably used in the clinical routine to measure CO. In this study we set to compare, for the first time, the TD-method with the eFM in a well-phenotyped aged real-world cohort of cardiological inpatients, showing a moderate correlation but large individual errors.

In the current study, only patients older than 60 years have been analysed, with this cut-off being previously defined by elderly population studies[16]. Most studies comparing TD and eFM focused on younger populations which typically suffered from PH or congenital heart disease.[1–3,17] While there are some studies investigating eFM in the subset of adult patients with PH, valvular heart diseases or heart failure [18–21], none of them have thoroughly investigated the old and the oldest-old population.[22–24] Similar trials comparing eFM and TD often did not report the formulas behind VO_2_ calculations due to their retrospective design.[17,20] An extensive analysis comparing the eFM and TD-method suggested that age is a major contributor to differences between the two methods, but did not further investigate the relevance of this observation.[20] As sarcopenia is frequent among elderly patients, especially in heart failure, VO_2_ assumptions based on young patient study-cohorts might be misleading.[16] Our study cohort differs from the derivation cohorts of LaF, De and Be concerning the estimation of VO_2_ as displayed in S1 Table.[1–3] Patients in the current study were older, had a higher BMI and suffered more frequently from a degenerative heart disease, such as aortic stenosis or mitral valve regurgitation, as a leading indication for cardiac catheterization. Importantly, there were no patients with cardiac shunts or congenital heart diseases.

### Correlation and agreement of thermodilution and indirect Fick method

Linear regression analysis showed only a modest correlation between TD and eFM among all three VO_2_ estimation formulas. The coefficient of determination in the current study was higher than described in two other studies with a similarly sized cohort of patients affected by PH[17,21], as well as in a large retrospective study of all-comers[20] (S7 Table). CI as assessed by the formulas of LaF and Be resulted in higher values than CI-TD in patients with low CI-states. This difference was already described in several studies.[5,17,18,25] In a similar way, previous works have shown CO derived by the LaF formula to be significantly lower as compared to TD[21] or direct FM[25]. Limits of agreement were narrower than described in previous similar-sized studies,[17] but still indicated a large margin of error.

Interestingly the correlation between VO_2_ –TD and estimated VO_2_ was weaker than for CI, showing the major impact of VO_2_ assumptions on the main hypothesis of the study.

A possible mode of action for the observed differences is probably aging-driven. While body weight might remain stable, the body composition changes with aging, showing an increase in fat mass and decrease in muscle mass.[26] As a higher muscle mass at same weight is associated with an increased VO_2,_[27] a possible explanation of the age-related CI differences could be related to body composition, with lower VO_2_/BSA in aged patients. Additionally, the LaF formula is the only formula that accounts for heart rate in a linear fashion.[1] This leads to two possible explanations for the observed differences with this formula a) children have physiologically higher heart rates than adults and b) a large proportion of our cohort received a beta blocker therapy.

### Confounders of CO measurement and estimation

In general, TD is preferred over eFM by guidelines in the assessment of PH and shows a better discriminatory power in the prediction of in-hospital and long-term mortality. On the one hand, the estimation of VO_2_ and the subsequent CI calculation by eFM have been described to be prone to error in the setting of congestive heart failure, PH or an abnormal body habitus[4,18,19,28]. On the other hand, certain conditions like severe valve regurgitations and a subsequent regurgitation-volume, as well as states of low-cardiac output have been reported to render TD unreliable. However, guidelines recommend the assessment of CI by TD even in patients with severe valve regurgitation, based on a study from Hoeper et al.[29,30]

Furthermore, the role of severe tricuspid regurgitation as a confounder for the TD remains a matter of debate. Several studies reported on systematic errors of TD measurement[22,23], leading to lower CI as compared to eFM.[20] More recent retrospective studies reported no influence of measured CI in patients with severe tricuspid regurgitation with primary or secondary PH.[17,21] In the current study severe tricuspid regurgitation led to significant discrepancies between TD and eFM when calculated by LaF, but not by De or Be. In contrast to previous reports[31], we did not observe a relevant impact of severe mitral valve regurgitation on the measurements. However, it must be pointed out that while the coefficient of correlation was worse when only investigating patients with severe tricuspid regurgitation, its presence alone was not a predictor for a difference greater than 20% between estimated and measured cardiac index. While this study is not designed to answer the question whether TD is applicable in patients with severe tricuspid regurgitation, we believe using an additional method to measure CO in such a cohort of patients to be a reasonable suggestion.

It has been reported that in states of low-CI, eFM tends to overestimate CI when compared to direct measurements.[17] In the present study, states of low CI were associated with lower CI values when comparing TD to eFM by the formulas of LaF and De but not Be. In a large retrospective study, CO, as assessed by TD, was superior to eFM in predicting mortality, while incorporating eFM-CO additionally to TD-CO lead to an improved risk stratification than TD alone in patients affected by cardiogenic shock.[20]

Finally, Narang et al.[18,19] and other groups[21] showed the inaccuracy of VO_2_ estimation in overweight patients and proposed to extend the classic eFM formulas with variables accounting for body composition. We used the caliper method proposed by Jackson and Pollock to estimate body fat percentage in a subgroup of our study population.[13,14] In contrast to those studies, body composition did not impact on the accuracy of CI-eFM in the investigated subgroup. However, the limited number of patients in the current analysis might have influenced our observation.

### Clinical relevance

CI measurements are essential to define the pathophysiology and to estimate prognostic outcomes in many cardiovascular diseases. Therefore, a precise and reproducible measurement is mandatory, especially in the evaluation of PH or valvular heart diseases. In Germany in 2016 valvular heart disease was most frequent among patients older than 60 years.[32] Additionally, while PH is still referred to as a young woman’s disease, the mean age for the first diagnosis has risen steadily and is currently at 65 years in Germany. A study investigating CI measurements with a focus on aged patients is therefore of great importance to implement and substantiate clinical guidelines.

Due to limited time and resources, many cath-labs use the eFM to assume CI. While the observed coefficient of determination of ~0.55 indicates a moderate correlation, it does not reflect the rate of error on an individual basis. In the current study, a large difference (20% or above) between TD-CI and eFM-CI was found among 27 to 41% of patients. Previous studies showed the similar large difference among 11 to 45% of patients, leading to misclassification of PH patients, as well as to a misdiagnosis of cardiogenic shock patients in 10–20% of cases. [17,25] Measuring CI through the acetylene rebreathing method might have been a valid alternative as this method has been shown to be reliable in various subsets of patient groups and is neither influenced by severe valvular disease nor states of cardiogenic shock. However, data focusing on aged patient cohorts are scarce.[29] In summary, the use of eFM-CI seems to fall short in the diagnostic workup of aged patients and should be interpreted cautiously. Available information on the advantage and disadvantage of TD-, iFM a direct Fick method are summarized in S8 Table.

In the setting of continuous monitoring of CI in the intensive care unit, more readily available variables like lactate, [33] ScVO_2_ [34]or other non-invasive CI measurement [35] play a major role in monitoring the course of therapy.

### Limitations

A major limitation of the study is related to be a retrospective single-center study. Therefore, measurements were performed within the routine clinical assessment. However, we believe these data to reflect a real-world setting better. Also, the quality of tracing has been assessed by two independent clinicians.

Another limitation is that the comparison of the indirect measurement of VO_2_ rather than direct one. Further investigations comparing direct Fick method and thermodilution in elderly patients is needed.

## Conclusion

TD and eFM only show moderate correlation with large individual errors in aged patients. The estimation of CI does not seem to be appropriate in the diagnostic workup of aged patients and should be interpreted cautiously. When precise hemodynamic assessment is necessary CI should not be estimated. Further multi-center studies in larger cohorts of patients are warranted.

## Supporting information

S1 File Additional statistical explanation. (DOCX)

S1 Table Empirical formulas for VO2 assumption. Abbreviations: SD denotes standard deviation; VO_2_, whole body oxygen consumption; NA, not available and BSA, body surface area.
(DOCX)

S2 Table Baseline hemodynamics and blood gas analysis values. Abbreviations: BPM denominates beats per minute; PAWP, pulmonary artery wedge pressure; PAP, pulmonary artery pressure; RAP, right atrial pressure; ABP, arterial blood pressure; LVP, left ventricular pressure; SAO_2_, arterial oxygen saturation; pO_2_, oxygen partial pressure, pCO_2,_ carbon dioxide partial pressure and SVO_2_ central venous saturation.
(DOCX)

S3 Table Correlation of cardiac index and whole-body oxygen consumption between thermodilution and indirect Fick method. Abbreviations: VO_2_ denominates whole-body oxygen consumption.
(DOCX)

S4 Table Correlation of thermodilution and indirect Fick method cardiac index in accordance to the presence of a severe or no severe tricuspid regurgitation. Abbreviations: TR denominates tricuspid regurgitation.
(DOCX)

S5 Table Predictors of a difference greater than 20% between estimated and measured cardiac index. Abbreviations: Lf denominates LaFarge; De, Dehmer; Be, Bergstra; TR, tricuspid regurgitation and BMI, body mass index.
(DOCX)

S6 Table Comparison of Bland-Altman plots between estimated and measured values of cardiac index and whole-body oxygen consumption within current literature. Abbreviations: LLA denominates lower limit of agreement; ULA upper limit of agreement; VO_2_, whole-body oxygen consumption; TD, thermodilution; ID, indicator-dylution eFM, estimated Fick method; Lf, LaFarge; De, Dehmer; Be, Bergstra; ?, unknown formula; eVO_2_ estimated whole-body oxygen consumption.
(DOCX)

S7 Table Comparison of correlation between estimated and measured values of cardiac index and whole-body oxygen consumption within current literature. Abbreviations: VO_2_ denominates whole-body oxygen consumption; TD, thermodilution; ID, indicator-dilution; eFM, estimated Fick method; Lf, LaFarge; De, Dehmer; Be, Bergstra; ?, unknown formula; eVO_2_ estimated whole-body oxygen consumption.
(DOCX)

S8 Table Comparison of advantages and disadvantages of cardiac output measurements methods. Abbreviations: eFM denominates estimated Fick method; CO, cardiac output; dFM, direct Fick method; TD, thermodilution method and 3D-TEE, three-dimensional transoesophageal echocardiography.
(DOCX)

## Abbreviations

ABP: arterial blood pressure
Be: Bergstras formula
BMI: Body mass index
Bpm: beats per minute
BSA: Body surface area
CI: Cardiac index
CO: Cardiac output
De: Dehmers formula
eFM: estimated Fick method
FM: Fick method
GFR: Glomerular filtration rate
HFmrEF: Heart failure with mid-range reduced ejection fraction
HFrEF: Heart failure with reduced ejection fraction
LaF: LaFarges formula
LVEF: Left ventricular ejection fraction
LVP: Left ventricular pressure
PAP: Pulmonary artery pressure
PAWP: Pulmonary artery wedge pressure
pCO2: carbon dioxide partial pressure
pO2: Oxygen partial pressure
RAP: Right atrial pressure
SaO2: Arterial oxygen saturation
SD: Standard deviation
SvO2: Central venous oxygen saturation
TAVI: Transcatheter aortic valve implantation
TD: thermodilution
VO2: Oxygen consumption
