# Supplementary material for: Thermodilution vs estimated Fick cardiac output measurement in an elderly cohort of patients: A single-centre experience
Source: PLoS One. 2019 Dec 20;14(12):e0226561. doi: 10.1371/journal.pone.0226561 (PMC6924680; doi:10.1371/journal.pone.0226561)
Supplement: S1 File — (DOCX) [file pone.0226561.s001.docx]

## S1 File: Additional statistical explanation

Statistical analysis

Receiver operating characteristic analysis was used to determine the area under the curve for continuous variables regarding the prediction of a difference equal or greater 20% for measured (thermodilution) or estimated (Fick method using formulas of either LaFarge, Dehmer or Bergstra) cardiac index. The predictive value of dichotomous / dichotomized variables regarding the above-mentioned difference was assessed using univariable logistic regression analyses.

A two-sided significance level of α 0.05 was defined appropriate to indicate statistical significance.
