# Supplementary material for: Thermodilution vs estimated Fick cardiac output measurement in an elderly cohort of patients: A single-centre experience
Source: PLoS One. 2019 Dec 20;14(12):e0226561. doi: 10.1371/journal.pone.0226561 (PMC6924680; doi:10.1371/journal.pone.0226561)
Supplement: S1 Table — Abbreviations: SD denotes standard deviation; VO2, whole body oxygen consumption; NA, not available and BSA, body surface area. (DOCX) [file pone.0226561.s002.docx]

**S1 Table: Empirical formulas for VO2 assumption**

|  | LaFarge | Dehmer | Bergstra |
| --- | --- | --- | --- |
| **Derivation year(s)** | 1961-1966 | 1982 | 1995 |
| **Patients** | 879 (♀ 41%) | 108 (♀ 36%) | 250 (♀ 43%) + 60 |
| **Age range (years),**  **(mean** + **SD)** | 3 – 40,  (13 + NA) | 21 – 75,  (50 + NA) | 1.2 -83.8,  (34.6 + 22.7) |
| **Reference-method** | Direct VO_2_ measurement | Thermo- und Dye-dilution method | Dye-dilution method |
| **Variables** | Age, gender, heart rate, BSA (DuBois). | BSA (DuBois) | Age, gender, BSA (DuBois) |

Abbreviations: SD denotes standard deviation; VO_2_, whole body oxygen consumption; NA, not available and BSA, body surface area.
