# Supplementary material for: Thermodilution vs estimated Fick cardiac output measurement in an elderly cohort of patients: A single-centre experience
Source: PLoS One. 2019 Dec 20;14(12):e0226561. doi: 10.1371/journal.pone.0226561 (PMC6924680; doi:10.1371/journal.pone.0226561)
Supplement: S2 Table — Abbreviations: BPM denominates beats per minute; PAWP, pulmonary artery wedge pressure; PAP, pulmonary artery pressure; RAP, right atrial pressure; ABP, arterial blood pressure; LVP, left ventricular pressure; SAO2, arterial oxygen saturation; pO2, oxygen partial pressure, pCO2, carbon dioxide partial pressure and SVO2 central venous saturation. (DOCX) [file pone.0226561.s003.docx]

**S2 Table: Baseline hemodynamics and blood gas analysis values**

| Hemodynamics | n = 155 |
| --- | --- |
| Heart rate (/bpm) | 75 ± 15.1 |
| Rhythm |  |
| Sinus | 88 (56.8) |
| Atrial fibrillation | 65 (41.9) |
| Other | 2 (1.2) |
| PAWP mean | 20.5 ± 8.4 |
| PAWP v-wave | 27.8 ± 13.5 |
| PAP systolic | 49.3 ± 17.8 |
| PAP diastolic | 21.9 ± 8.5 |
| PAP mean | 32.8 ± 11.8 |
| RAP mean | 11.1 ± 6.3 |
| ABP systolic | 144.9 ± 32.1 /142 |
| ABP diastolic | 66.9 ±13.9 /142 |
| ABP mean | 96.4 ± 18.8 /142 |
| LVP systolic | 161.6 ± 38.1 /137 |
| LVP diastolic | -2.99 ± 7.7 /137 |
| LVP enddiastolic | 19.6 ± 7.1 /137 |
| Pulmonary hypertension |  |
| Precapillary pulmonary hypertension | 11 (7.2)/153 |
| Postcapillary pulmonary hypertension | 97 (63.4)/153 |
| Blood gas analysis |  |
| Temperatur | 36.7 ± 0.4 |
| Haemoglobin mean | 12.2 ± 1.9 |
| Arterial |  |
| SAO_2_ (%) | 94.7 ± 4.1 |
| Haemoglobin (mg/dl) | 12.2 ± 2.0 |
| pO_2_ (mmHg) | 82.1 ± 25.0 |
| pCO_2_ (mmHg) | 38.9 ± 8.1 |
| Base Excess | 1.3 ± 3.8 |
| pH | 7.43 ± 0.04 |
| Oxygen capacity (g/dl) | 15.5 ± 2.5 |
| Mived venous |  |
| SVO_2_ (%) | 62.0 ± 8.8 |
| Haemoglobin (mg/dl) | 12.3 ± 1.9 |
| pO_2_ (mmHg) | 35.3 ± 4.5 |
| pCO_2_ (mmHg) | 43.9 ± 7.1 |
| Base Excess | 2.7 ± 3.7 |
| pH | 7.40 ± 0.38 |
| Oxygen capacity (g/dl) | 10.2 ± 2.5 |

Abbreviations: BPM denominates beats per minute; PAWP, pulmonary artery wedge pressure; PAP, pulmonary artery pressure; RAP, right atrial pressure; ABP, arterial blood pressure; LVP, left ventricular pressure; SAO_2_, arterial oxygen saturation; pO_2_, oxygen partial pressure, pCO_2,_ carbon dioxide partial pressure and SVO_2_ central venous saturation.
