# Supplementary material for: Thermodilution vs estimated Fick cardiac output measurement in an elderly cohort of patients: A single-centre experience
Source: PLoS One. 2019 Dec 20;14(12):e0226561. doi: 10.1371/journal.pone.0226561 (PMC6924680; doi:10.1371/journal.pone.0226561)
Supplement: S3 Table — Abbreviations: VO2 denominates whole-body oxygen consumption. (DOCX) [file pone.0226561.s004.docx]

**S3 Table: Correlation of cardiac index and whole-body oxygen consumption between thermodilution and indirect Fick method**

|  | **Cardiac index** | |  | **VO_2_** | |
| --- | --- | --- | --- | --- | --- |
| **Method** | **r² (95% CI)** | **p** |  | **r² (95% CI)** | **p** |
| Lafarge | 0.53 (0.42-0.63) | <0.001 |  | 0.38 (0.25-0.49) | <0.001 |
| Dehmer | 0.54 (0.42-0.64) | <0.001 |  | 0.39 (0.27-0.51) | <0.001 |
| Bergstra | 0.57 (0.45-0.66) | <0.001 |  | 0.40 (0.28-0.52) | <0.001 |

Abbreviations: VO_2_ denominates whole-body oxygen consumption.
