# Supplementary material for: Thermodilution vs estimated Fick cardiac output measurement in an elderly cohort of patients: A single-centre experience
Source: PLoS One. 2019 Dec 20;14(12):e0226561. doi: 10.1371/journal.pone.0226561 (PMC6924680; doi:10.1371/journal.pone.0226561)
Supplement: S4 Table — Abbreviations: TR denominates tricuspid regurgitation. (DOCX) [file pone.0226561.s005.docx]

**S4 Table: Correlation of thermodilution and indirect Fick method cardiac index in accordance to the presence of a severe or no severe tricuspid regurgitation**

|  | **mild-moderate TR**  **(n=132)** | |  | **Severe TR**  **(n=23)** | |
| --- | --- | --- | --- | --- | --- |
| **Method** | **r² (95% CI)** | **p** |  | **r² (95% CI)** | **p** |
| Lafarge | 0.67 (0.57-0.75) | <0.001 |  | 0.38 (0.07-0.67) | 0.002 |
| Dehmer | 0.62 (0.50-0.71) | <0.001 |  | 0.59 (0.27-0.80) | <0.001 |
| Bergstra | 0.65 (0.54-0.74) | <0.001 |  | 0.57 (0.25-0.79) | <0.001 |

Abbreviations: TR denominates tricuspid regurgitation.
