# Supplementary material for: Thermodilution vs estimated Fick cardiac output measurement in an elderly cohort of patients: A single-centre experience
Source: PLoS One. 2019 Dec 20;14(12):e0226561. doi: 10.1371/journal.pone.0226561 (PMC6924680; doi:10.1371/journal.pone.0226561)
Supplement: S5 Table — Abbreviations: Lf denominates LaFarge; De, Dehmer; Be, Bergstra; TR, tricuspid regurgitation and BMI, body mass index. (DOCX) [file pone.0226561.s006.docx]

**S5 Table: Predictors of a difference greater than 20% between estimated and measured cardiac index**

| **Method** | **OR (95% CI)**  **20% difference Lf**  **(n=63)** | **p** | **OR (95% CI)**  **20% difference De**  **(n=41)** | **p** | **OR (95% CI)**  **20% difference Be**  **(n=56)** | **p** |
| --- | --- | --- | --- | --- | --- | --- |
| Severe TR | 0.74 (0.29-1.86) | 0.518 | 1.58 (0.62-4.07) | 0.340 | 1.42 (0.58-3.49) | 0.443 |
| Cardiac Index <2.2 l/min/m² | 0.73 (0.38-1.4) | 0.351 | 1.41 (0.69-2.89) | 0.350 | **2.55 (1.30-4.99)** | **0.006** |
| Female sex | **2.58 (1.33-5.00)** | **0.005** | 1.46 (0.71-3.00) | 0.302 | 1.33 (0.68-2.59) | 0.394 |
| Age per year | 1.03 (0.99-1.08) | 0.185 | 1.04 (0.99-1.10) | 0.127 | 1.02 (0.97-1.07) | 0.455 |
| Age ≥78 years | **2.24 (1.2-4.35)** | **0.017** | 1.70 (0.82-3.49) | 0.152 | 1.41 (0.72-2.75) | 0.312 |
| Heart rate (bpm) | 0.99 (0.97-1.01) | 0.470 | 0.99 (0.97-1.02) | 0.560 | 1.00 (0.98-1.03) | 0.756 |
| Heart rate ≥100 bpm | 0.91 (0.28-2.91) | 0.867 | 0.48 (0.10-2.27) | 0.480 | 1.58 (0.50-5.00) | 0.435 |
| No sinus rhythm | 1.21 (0.64-2.31) | 0.560 | 1.19 (0.58-2.43) | 0.639 | 1.09 (0.57-2.12) | 0.789 |
| BMI ≥30 kg/m² | 0.64 (0.31-1.30) | 0.216 | 0.54 (0.24-1.25) | 0.149 | 1.09 (0.54-2.21) | 0.812 |
| BMI ≥35 kg/m² | 0.71 (0.21-2.47) | 0.593 | 3.09 (0.94-10.1) | 0.064 | 1.86 (0.57-6.07) | 0.304 |
| Body fat ≥30 % | 0.78 (0.37-1.62) | 0.501 | 0.67 (0.29-1.57) | 0.357 | 1.00 (0.47-2.14) | 1.000 |

Abbreviations: Lf denominates LaFarge; De, Dehmer; Be, Bergstra; TR, tricuspid regurgitation and BMI, body mass index.
