# Supplementary material for: Thermodilution vs estimated Fick cardiac output measurement in an elderly cohort of patients: A single-centre experience
Source: PLoS One. 2019 Dec 20;14(12):e0226561. doi: 10.1371/journal.pone.0226561 (PMC6924680; doi:10.1371/journal.pone.0226561)
Supplement: S6 Table — Abbreviations: LLA denominates lower limit of agreement; ULA upper limit of agreement; VO2, whole-body oxygen consumption; TD, thermodilution; ID, indicator-dylution eFM, estimated Fick method; Lf, LaFarge; De, Dehmer; Be, Bergstra; ?, unknown formula; eVO2 estimated whole-body oxygen consumption. (DOCX) [file pone.0226561.s007.docx]

**S6 Table: Comparison of Bland-Altman plots between estimated and measured values of cardiac index and whole-body oxygen consumption within current literature**

|  | **n** | **Age** | **Cardiac index**  **Bias (LLA-ULA)** | **Cardiac output**  **Bias (LLA-ULA)** | **VO²**  **Bias (LLA-ULA)** | **Patient population** |
| --- | --- | --- | --- | --- | --- | --- |
| Kresoja et al.  TD and eFM (Lf)  TD and eFM (De)  TD and eFM (Be) | 155 | 75.1 ± 6.8 | 0.22 (-0.64 to 1.09)  -0.15 (-1.07 to 0.77)  -0.42 (-1.38 - 0.53) | 0.35 (-1.3-2.0)  -0.34 (-2.1-1.5)  -0.89 (-2.8-1.0) | 20 (-64-104)  -14 (-98-70)  -41 (-126-43) | Geriatric all comers |
| Li et al.[1]  VO_2_ and eVO_2_ (Lf)  VO_2_ and eVO_2_ (Lf) | 75  51 | 4.9 ± 5.5  2.9 ± 3.5 | -  - | -  - | 4.5 (-76.9-85.9)  -15.4 (-132.5-101.6) | Congenital heart disease |
| Schmitz et al.[2]  VO_2_ and eVO_2_ (Lf) | 52 | 6.9 | - | - | 8.9 (-39.4-57.2) | Congenital heart disease |
| Rutledge et al.[3]  VO_2_ and eVO_2_ (Lf) | 75 | 0.13 to 24  all  <3 years  >3 years | -  -  - | -  -  - | 33 (-56-123)  55 (-42-153)  11 (-39-61) | Congenital heart disease |
| Bergstra et al.[4] | 60 | 40 ± 22 | - | - | 2 (-45-49) | Congenital and acquired heart disease |
| Wolf et al.[5]  VO_2_ and eVO_2_ (Lf) VO_2_ and eVO_2_ (Be) | 57 | 52 | -  - | -  - | 8 (-41-57)  -16 (-60-30) | All comers |
| Chase et al.[6]  VO_2_ and eVO_2_ (Lf)  VO_2_ and eVO_2_ (De)  VO_2_ and eVO_2_ (Be) | 44 | 65 ± 10.7 | -  -  - | -  -  - | -3 (-35-30)  11 (-29-50)  21 (-21-64) | Systolic heart failure |
| Fares et al. [7]  TD and eFM (?) | 213 | 54 ± 13 | - | -0.39 (-4.44-3.66) | - | Pulmonary hypertension |
| Alkhodair et al.[8]  TD and eFM (Lf) | 168 | 63 ± 15 |  | 0.6 (-3.3-2.1) |  | Pulmonary hypertension |
| Opotowsky et al.[9]  TD and eFM (?) | 12.232 | 66.2 ± 10.0 | -0.2 (-1.31-1.27) | **-** | **-** | Veterans hospital all-comers |

Abbreviations: LLA denominates lower limit of agreement; ULA upper limit of agreement; VO_2_, whole-body oxygen consumption; TD, thermodilution; ID, indicator-dylution eFM, estimated Fick method; Lf, LaFarge; De, Dehmer; Be, Bergstra; ?, unknown formula; eVO_2_ estimated whole-body oxygen consumption.

**References**

1. Li J, Bush A, Schulze-Neick I, Penny DJ, Redington AN, Shekerdemian LS. Measured versus estimated oxygen consumption in ventilated patients with congenital heart disease. The validity of predictive equations. Crit Care Med. 2003; 31: 1235–1240. doi: 10.1097/01.CCM.0000060010.81321.45.

2. Schmitz A, Kretschmar O, Knirsch W, Woitzek K, Balmer C, Tomaske M, et al. Comparison of calculated with measured oxygen consumption in children undergoing cardiac catheterization. Pediatr Cardiol. 2008; 29: 1054–1058. doi: 10.1007/s00246-008-9248-6.

3. Rutledge J, Bush A, Shekerdemian L, Schulze-Neick I, Penny D, Cai S, et al. Validity of the LaFarge equation for estimation of oxygen consumption in ventilated children with congenital heart disease younger than 3 years--a revisit. Am Heart J. 2010; 160: 109–114. doi: 10.1016/j.ahj.2010.04.003.

4. Bergstra A, van Dijk RB, Hillege HL, Lie KI, Mook GA. Assumed oxygen consumption based on calculation from dye dilution cardiac output. An improved formula. Eur Heart J. 1995; 16: 698–703.

5. Wolf A, Pollman MJ, Trindade PT, Fowler MB, Alderman EL. Use of assumed versus measured oxygen consumption for the determination of cardiac output using the Fick principle. Cathet Cardiovasc Diagn. 1998; 43: 372–380.

6. Chase PJ, Davis PG, Wideman L, Starnes JW, Schulz MR, Bensimhon DR. Comparison of Estimations Versus Measured Oxygen Consumption at Rest in Patients With Heart Failure and Reduced Ejection Fraction Who Underwent Right-Sided Heart Catheterization. Am J Cardiol. 2015; 116: 1724–1730. doi: 10.1016/j.amjcard.2015.08.051.

7. Fares WH, Blanchard SK, Stouffer GA, Chang PP, Rosamond WD, Ford HJ, et al. Thermodilution and Fick cardiac outputs differ. Impact on pulmonary hypertension evaluation. Can Respir J. 2012; 19: 261–266. doi: 10.1155/2012/261793.

8. Alkhodair A, Tsang MYC, Cairns JA, Swiston JR, Levy RD, Lee L, et al. Comparison of thermodilution and indirect Fick cardiac outputs in pulmonary hypertension. Int J Cardiol. 2018; 258: 228–231. doi: 10.1016/j.ijcard.2018.01.076.

9. Opotowsky AR, Hess E, Maron BA, Brittain EL, Barón AE, Maddox TM, et al. Thermodilution vs Estimated Fick Cardiac Output Measurement in Clinical Practice. An Analysis of Mortality From the Veterans Affairs Clinical Assessment, Reporting, and Tracking (VA CART) Program and Vanderbilt University. JAMA Cardiol. 2017; 2: 1090–1099. doi: 10.1001/jamacardio.2017.2945.
