# Supplementary material for: Thermodilution vs estimated Fick cardiac output measurement in an elderly cohort of patients: A single-centre experience
Source: PLoS One. 2019 Dec 20;14(12):e0226561. doi: 10.1371/journal.pone.0226561 (PMC6924680; doi:10.1371/journal.pone.0226561)
Supplement: S7 Table — Abbreviations: VO2 denominates whole-body oxygen consumption; TD, thermodilution; ID, indicator-dilution; eFM, estimated Fick method; Lf, LaFarge; De, Dehmer; Be, Bergstra; ?, unknown formula; eVO2 estimated whole-body oxygen consumption. (DOCX) [file pone.0226561.s008.docx]

**S7 Table: Comparison of linear correlation between estimated and measured values of cardiac index and whole-body oxygen consumption within current literature**

|  | **n** | **Age** | **Cardiac index/output**  **r² (95% CI)** | **VO_2_**  **r² (95% CI)** | **Reference method** | **Patient population** |
| --- | --- | --- | --- | --- | --- | --- |
| Kresoja et al.  TD and eFM (Lf)  TD and eFM (De)  TD and eFM (Be) | 155 | 75.1 ± 6.8 | 0.53 (0.42-0.63)  0.54 (0.42-0.64)  0.57 (0.45-0.66) | 0.38 (0.25-0.49)  0.39 (0.27-0.51)  0.40 (0.28-0.52) | Thermodlution | Geriatric all comers |
| Schmitz et al.[1]  VO2 and eVO2 (Lf) | 52 | 6.9 | - | 0.81 (0.69-0.89) | Metabolic Monitor | Congenital heart disease |
| Wolf et al.[2]  VO_2_ and eVO_2_ (Lf) TD and eVO_2_ (Be) | 57 | 52 | -  - | 0.35 (0.15-0.55)  0.29 (0.10-0.49) | Metabolic monitor | All comers |
| Fares et al. [3]  TD and eFM (?) | 213 | 54 ± 13 | 0.30 (0.20-0.40) | - | Thermodilution | Pulmonary hypertension |
| Alkhodair et al.[4]  TD and eFM (Lf) | 168 | 63 ± 15 | 0.38 (0.26-0.49) | - | Thermodilution | Pulmonary hypertension |
| Opotowsky et al.[5]  TD and eFM (?) | 12.232 | 66.2 ± 10.0 | 0.42 (0.41-0.44) | - | Thermodilution | Veterans hospital all-comers |
| Hillis et al. [6]  TD and eFM (Lf)  ID and eFM (Lf) | 252  556 | 50 ± 11 | 0.79 (0.74-0.83)  0.83 (0.80-0.85) | -  - | Thermodilution and indocyanine green | All comers |
| Fakler et al.[7]  VO_2_ and eVO_2_ | 143 | 11.3 ± 8.1 | - | 0.38 (0.25-0.50) | Douglas bag | Congenital heart disease |
| Narang et al.[8]  VO_2_ and eVO_2_ (Lf)  VO_2_ and eVO_2_ (De)  VO_2_ and eVO_2_ (Be) | 535 | 55 ± 13.5 | -  -  - | 0.24 (0.18-0.30)  0.32 (0.25-0.38)  0.33 (0.26-0.39) | Douglas bag | All comers |

Abbreviations: VO_2_ denominates whole-body oxygen consumption; TD, thermodilution; ID, indicator-dilution; eFM, estimated Fick method; Lf, LaFarge; De, Dehmer; Be, Bergstra; ?, unknown formula; eVO_2_ estimated whole-body oxygen consumption.

**References**

1. Schmitz A, Kretschmar O, Knirsch W, Woitzek K, Balmer C, Tomaske M, et al. Comparison of calculated with measured oxygen consumption in children undergoing cardiac catheterization. Pediatr Cardiol. 2008; 29: 1054–1058. doi: 10.1007/s00246-008-9248-6.

2. Wolf A, Pollman MJ, Trindade PT, Fowler MB, Alderman EL. Use of assumed versus measured oxygen consumption for the determination of cardiac output using the Fick principle. Cathet Cardiovasc Diagn. 1998; 43: 372–380.

3. Fares WH, Blanchard SK, Stouffer GA, Chang PP, Rosamond WD, Ford HJ, et al. Thermodilution and Fick cardiac outputs differ. Impact on pulmonary hypertension evaluation. Can Respir J. 2012; 19: 261–266. doi: 10.1155/2012/261793.

4. Alkhodair A, Tsang MYC, Cairns JA, Swiston JR, Levy RD, Lee L, et al. Comparison of thermodilution and indirect Fick cardiac outputs in pulmonary hypertension. Int J Cardiol. 2018; 258: 228–231. doi: 10.1016/j.ijcard.2018.01.076.

5. Opotowsky AR, Hess E, Maron BA, Brittain EL, Barón AE, Maddox TM, et al. Thermodilution vs Estimated Fick Cardiac Output Measurement in Clinical Practice. An Analysis of Mortality From the Veterans Affairs Clinical Assessment, Reporting, and Tracking (VA CART) Program and Vanderbilt University. JAMA Cardiol. 2017; 2: 1090–1099. doi: 10.1001/jamacardio.2017.2945.

6. Hillis LD, Firth BG, Winniford MD. Analysis of factors affecting the variability of Fick versus indicator dilution measurements of cardiac output. Am J Cardiol. 1985; 56: 764–768.

7. Fakler U, Pauli C, Hennig M, Sebening W, Hess J. Assumed oxygen consumption frequently results in large errors in the determination of cardiac output. J Thorac Cardiovasc Surg. 2005; 130: 272–276. doi: 10.1016/j.jtcvs.2005.02.048.

8. Narang N, Thibodeau JT, Levine BD, Gore MO, Ayers CR, Lange RA, et al. Inaccuracy of estimated resting oxygen uptake in the clinical setting. Circulation. 2014; 129: 203–210. doi: 10.1161/CIRCULATIONAHA.113.003334.
