# Supplementary material for: Thermodilution vs estimated Fick cardiac output measurement in an elderly cohort of patients: A single-centre experience
Source: PLoS One. 2019 Dec 20;14(12):e0226561. doi: 10.1371/journal.pone.0226561 (PMC6924680; doi:10.1371/journal.pone.0226561)
Supplement: S8 Table — Abbreviations: eFM denominates estimated Fick method; CO, cardiac output; dFM, direct Fick method; TD, thermodilution method and 3D-TEE, three-dimensional transoesophageal echocardiography. (DOCX) [file pone.0226561.s009.docx]

**S8 Table: Comparison of advantages and disadvantages of cardiac output measurements methods**

|  | **Thermodilution method** | **Indirect Fick method** | **Direct Fick method** |
| --- | --- | --- | --- |
| **Mortality prediction** | Higher predictive value for 90 and 360 days mortality as comparted to eFM[1] | Inferior to eFM[1] | ? |
| **Pulmonary hypertension** | Recommended by guidelines[2] comparable to direct Fick method[3] | Tends to overestimate[4]/undererstimate[5] CO | Recommended by guidelines[2] comparable to thermodilution[3] |
| **Low Cardiac Output** | Superior to eFM[1]; overestimation compared to dFM if dye dilution is used[6] | Inferior to eFM[1], tends to overestimate as compared to TD[1,4] | Comparable to TD method[3,6] |
| **Aortic stenosis** | Poor correlation in the prediction of severe aortic stenosis as compared to 3D-TEE (Sens/Spec 81/42 %)[7] | ? | Poor correlation in the prediction of severe aortic stenosis as compared to 3D-TEE (Sens/Spec 97/53 %)[7] |
| **Severe tricuspid regurgitation** | Conflicting data on underestimation[8,9] or adequate estimation as compared to dFM[3], recommended by Guidelines[2] | No relevant difference as compared to TD[4] | Gold standard and recommended by guidelines[2,3] |
| **Clinical availability** | Modest investigator experience required | Low investigator experience | High-investigator and team experience required |
| **Cost** | Low | Very low | Moderate |

Abbreviations: eFM denominates estimated Fick method; CO, cardiac output; dFM, direct Fick method; TD, thermodilution method and 3D-TEE, three-dimensional transoesophageal echocardiography

**References**

1. Opotowsky AR, Hess E, Maron BA, Brittain EL, Barón AE, Maddox TM, et al. Thermodilution vs Estimated Fick Cardiac Output Measurement in Clinical Practice. An Analysis of Mortality From the Veterans Affairs Clinical Assessment, Reporting, and Tracking (VA CART) Program and Vanderbilt University. JAMA Cardiol. 2017; 2: 1090–1099. doi: 10.1001/jamacardio.2017.2945.

2. Galiè N, Humbert M, Vachiery J-L, Gibbs S, Lang I, Torbicki A, et al. 2015 ESC/ERS Guidelines for the diagnosis and treatment of pulmonary hypertension. The Joint Task Force for the Diagnosis and Treatment of Pulmonary Hypertension of the European Society of Cardiology (ESC) and the European Respiratory Society (ERS): Endorsed by: Association for European Paediatric and Congenital Cardiology (AEPC), International Society for Heart and Lung Transplantation (ISHLT). Eur Heart J. 2016; 37: 67–119. doi: 10.1093/eurheartj/ehv317.

3. Hoeper MM, Maier R, Tongers J, Niedermeyer J, Hohlfeld JM, Hamm M, et al. Determination of cardiac output by the Fick method, thermodilution, and acetylene rebreathing in pulmonary hypertension. Am J Respir Crit Care Med. 1999; 160: 535–541. doi: 10.1164/ajrccm.160.2.9811062.

4. Fares WH, Blanchard SK, Stouffer GA, Chang PP, Rosamond WD, Ford HJ, et al. Thermodilution and Fick cardiac outputs differ. Impact on pulmonary hypertension evaluation. Can Respir J. 2012; 19: 261–266. doi: 10.1155/2012/261793.

5. Alkhodair A, Tsang MYC, Cairns JA, Swiston JR, Levy RD, Lee L, et al. Comparison of thermodilution and indirect Fick cardiac outputs in pulmonary hypertension. Int J Cardiol. 2018; 258: 228–231. doi: 10.1016/j.ijcard.2018.01.076.

6. Hillis LD, Firth BG, Winniford MD. Analysis of factors affecting the variability of Fick versus indicator dilution measurements of cardiac output. Am J Cardiol. 1985; 56: 764–768.

7. Gertz ZM, Raina A, O'Donnell W, McCauley BD, Shellenberger C, Kolansky DM, et al. Comparison of invasive and noninvasive assessment of aortic stenosis severity in the elderly. Circ Cardiovasc Interv. 2012; 5: 406–414. doi: 10.1161/CIRCINTERVENTIONS.111.967836.

8. Kadota LT. Theory and application of thermodilution cardiac output measurement. A review. Heart Lung. 1985; 14: 605–616.

9. Cigarroa RG, Lange RA, Williams RH, Bedotto JB, Hillis LD. Underestimation of cardiac output by thermodilution in patients with tricuspid regurgitation. Am J Med. 1989; 86: 417–420.
